# Growth and metal uptake of spinach with application of co-compost of cat manure and spent coffee ground

**DOI:** 10.1016/j.heliyon.2020.e05086

**Published:** 2020-09-28

**Authors:** Siti Nurathirah Kamaliah Mohd Noor Keeflee, Wan Nur Azra Wan Mohd Zain, Muhammad Nuruddin Mohd Nor, Nurul’ Ain Jamion, Soon Kong Yong

**Affiliations:** a Soil Assessment and Remediation Research Group, Faculty of Applied Sciences, Universiti Teknologi MARA, 40450 Shah Alam, Selangor, Malaysia; b Faculty of Plantation and Agrotechnology, Universiti Teknologi MARA, 77300 Merlimau, Jasin, Melaka, Malaysia; c School of Chemistry and Environment, Faculty of Applied Sciences, Universiti Teknologi MARA, Negeri Sembilan Branch, Kuala Pilah Campus, 72000 Kuala Pilah, Negeri Sembilan, Malaysia; d Research Centre for Sustainability Science & Governance (SGK), Institute for Environment and Development (LESTARI), Universiti Kebangsaan Malaysia, 43600 UKM Bangi, Selangor, Malaysia

**Keywords:** Co-compost, *Spinacia oleracea*, Cat manure, Heavy metal, Spent coffee ground, Fertilizer, Materials science, Chemistry, Food science, Agricultural science, Environmental science

## Abstract

Cat manure (CM) possesses high level of nutrients for growing food crop. However, animal manure may contain toxic elements that may contaminate food crop. Spent coffee ground (SCG) may be used to reduce mobility of heavy metals and reduce crop uptake. In this study, SCG was composted with CM for 31 days to produce a co-compost (SCG-CM) for growing spinach (*Spinacia oleracea*). The growth rate of spinach was assessed until its maturity, and the metal uptake of spinach shoot was determined thereafter using inductively coupled plasma-optical emission spectroscopy (ICP-OES). The effect of soil treatment with SCG-CM on the height and elemental composition of spinach were compared with that of chicken manure compost (CMC). The prepared composts were primarily organic matter (72.9–81.4 % w/w) with the rest are ash (13.3–23.4 % w/w) and moisture (1.2–2.6 % w/w). Zinc content in SCG-CM (1261 ± 0.1 mg/kg) is significantly higher than that of soil and CMC (p < 0.05) and has exceeded the maximum permissible limit set by European Union Standard (2002) and the Malaysian Compost Quality Standard and Guidelines (2000). Matured spinach reached maximum plant height after 33 days. The amendment of SCG-CM significantly increased the height of spinach (32 ± 6 cm) compared to that of CMC (13 ± 1 cm) (p < 0.05). However, contents of Zn, Cu, Pb and Cd were not increased for spinach grown in the SCG-CM-amended soil, and the level of those elements are below permissible limit set by the Malaysian Food Act 1983 and Food Regulations 1985. This study shows that SCG-CM is effective in improving yield without causing accumulation of toxic trace elements in spinach.

## 1 Introduction

Animal manure has been used to produce co-compost for increasing nutrient in soil (Hu et al., 2019). Conventionally, manures from poultry and ruminants (i.e, goat, sheep, cow) are composted as it is or combined with sawdust to produce organic fertilizer. However, these animals are not common household pets especially in the urbanized residential area. Cat is the most popular household pet animal in Malaysia as it is not restricted by religion. Cat manure (CM) is usually treated with clay-based cat litter (i.e., bentonite) in which the bad odour from CM is immobilized. In California, USA, an estimated 1.2 million tons of CM was disposed into the environment each year (Torrey and Yolken, 2013). Cat manure is particularly valuable due to its high nutrient content especially nitrogen, and it has great potential as fertilizer. Even though application of CM is gaining popularity (Xinhua, 2018), it's use for producing food crop has always been a taboo due to the potential contamination of protozoan parasite (i.e., *Taxoplasma gondii*) (Meerburg et al., 2006). Moreover, CM may also be contaminated with heavy metals due to possible ingestion of contaminated animal feed (Jiang et al., 2011). Application of the contaminated manure may cause migration to food crop and endangers human life if ingested.

Spent coffee ground (SCG) is produced in large quantity due to the increasing demand of coffee beverage from the growing global population (Quadra et al., 2020). Contrary to CM, application of SCG in agriculture is more accepted and it has been used for producing livestock feed (Choi et al., 2019) and organic fertilizer (Murthy and Madhava Naidu, 2012). Although direct application of fresh or composted SCG may introduce phytotoxic polyphenol compounds and suppress crop growth (Cervera-Mata et al., 2020), polyphenols may act as a chelating agent for immobilizing heavy metals. In fact, SCG has higher cadmium (Cd) maximum sorption capacity than that of zeolite (Kim and Kim, 2020). Co-composting of CM with SCG may alleviate migration of heavy metals from CM to food crop. Moreover, the additional animal manure in SCG helps reduced plant toxicity and improved plant growths (Yamane et al., 2014). Cat manure may provide additional nitrogen source to supplement the loss of nitrogen from SCG during composting process.

Composting of SCG may reduce the pH and this may increase the risk of mobilization of heavy metals from CM. Moreover, crop such as spinach (*Spinacia oleracea*) may hyperaccumulate heavy metals migrated from CM and further increase the health risk of the crop. To the best of our knowledge, the potential of CM as fertilizer for growing food crop and the accumulation of heavy metals in food crop has never been studied. This study aims to produce a novel co-compost (SCG-CM) by employing SCG and CM as source of carbon and nitrogen, respectively. The effect of applying SCG-CM on the growth and metal uptake of spinach were investigated. The contents for Zn, Cu, Pb and Cd in SCG-CM were compared with the permissible limits of the European Union Standard (2002) and the Malaysian Compost Quality Standard and Guidelines (2000). The suitability of spinach for human consumption were evaluated in terms of wet weight contents of Zn, Cu, Pb and Cd in spinach against the permissible limit set by the Malaysian Food Act 1983 and Food Regulations 1985.

## 2 Material and methods

### 2.1 Collection and preparation of CM and SCG

About 50 g of fresh CM was collected from a cat potty. Hair and cat litter particles were carefully removed from the air-dried CM using tweezers. The SCG was collected from a Starbucks Café at Seksyen 9, Shah Alam, Malaysia and stored in a clear, sealed plastic bag. For the purpose of comparison, chicken manure was collected from a farm at Klang, Selangor, Malaysia for producing CMC. All manures were immediately pasteurized by heating at about 80 °C for 60 min using an oven and then air-dried for 3 days at room temperature (ca. 25–35 °C). For the preparation of composts, CM and SCG were mixed at 1:3 weight ratio. The chicken manure was mixed with hardwood sawdust at 1:1 weight ratio and was composted at conditions similar to that for SCG-CM. A perforated and sterilized plastic container (dimension: 40 cm width х 60 cm length х 15 cm height) was lined with a piece of sterilized cotton cloth. About 100 g of manure was placed at the center of the container and was covered with a sterilized cotton cloth. It was then heaped up to a height of 15 cm. For the entire duration of composting, 10 ml of sterilized tap water was sprayed daily to the compost in order to maintain the moisture content. Composts was kept in a well-ventilated indoor setting at 28 ± 4 °C. For the first 15 days of the composting period, the mixture of composting materials was turned over every other day to maintain optimal composting conditions. Samples were collected randomly, and the temperature and pH of the compost was measured using thermometer pH meter, respectively, at 11 am daily for 40 days. Soil samples were purchased from a landscaping nursery located at Shah Alam, Selangor, Malaysia and were used without further treatment.

### 2.2 Determination of soil pH and electric conductivity

Six grams of calcium chloride was weighed and dissolved in 5.0 L of water. Then 2.0 g of samples from the pristine and amended soil was stirred with 5.0 mL of calcium chloride solution. After that, the solution pH was measured using a calibrated pH meter (LAB 850 BNC SET) (Salleh et al., 2017). The pH of the compost was measured daily for the entire duration of the composting process (i.e., 29 days). As for the electrical conductivity (EC), a calibrated EC meter (Eutech CON 510) was used to measure the EC of the matured composts and soil samples.

### 2.3 Determination of moisture content, organic matter and ash content by gravimetry

Five grams of air-dried SGC-CM, CMC, and soil samples were weighed to determine the moisture content, organic matter (OM) and ash content. All samples were placed in an oven for 24 h at 70 °C, and the final weight was recorded. Then, the oven-dried samples were placed into a desiccator before they were combusted in a furnace at 550 °C for 24 h. Then, the final weight was recorded using a calibrated analytical balance. The moisture content, OM, and ash content were calculated based on the wet weight of samples according to Eqs. (1), (2), and (3), respectively (Yong et al., 2015).

(1) $$Moisture\;content\;\left(\%\;w/w\;wet\;basis\right)\;=\;\frac{m_{a}-m_{o}}{m_{a}}\times 100$$

(2) $$OM\;content\;\left(\%\;w/w\;wet\;basis\right)\;=\;\frac{m_{o}-m_{f}}{m_{a}}\times 100$$

(3) $$Ash\;content\;\left(\%\;w/w\;wet\;basis\right)\;=\;\frac{m_{f}}{m_{a}}\times 100$$

where *m*_a_, *m*_o_ and *m*_f_ are weight of air-dried, oven-dried, and combusted samples, respectively.

### 2.4 Pot experiment

Spinach seeds were soaked in warm water overnight. Three seeds were placed in polyester bags (dimension: 20 cm × 20 cm × 25 cm) containing about 700 mL of soil (control), SCG-CM-amended soil (5 % w/w wet basis), and CMC-amended soil (5 % w/w wet basis). Each polyester bag was sprayed with about 20 mL of tap water daily. The daily plant growth was determined by counting the number of leaves and by measuring the height of stem (cm), width (cm) and length (cm) of leaves using a measuring tape. Stem height was measure from base to the top of the spinach. The width of leaves was measured from end-to-end between the widest lobes of the lamina perpendicular to the lamina mid-rib. The length of leaves was measured on each leaf from the pointy part at one end of the leaf to the point where the leaf joins the stalk at the other end. Compost-amended soils were collected and oven-dried at 80 °C for 24 h and kept in a desiccator. Spinach samples were harvested after 29 days. The shoots and the roots were separated and washed with tap water and rinsed with deionized water. The wet weight of spinach was recorded with a calibrated analytical balance. The plant samples were oven-dried at 80 °C for 24 h, and the dry weight were recorded by using a calibrated analytical balance.

### 2.5 Digestion of samples

One gram of oven-dried samples was placed into a 50 mL beaker, and then added with 10 mL of 65 % nitric acid and 4 mL of 30 % hydrogen peroxide (Pequerul et al., 1993). Then, the mixture was placed on a hot plate and heated vigorously at 80 °C. After that, 3mL of 65% nitric acid was added continuously until a colourless solution was obtained. The solution was allowed to cool and then filtered with Whatman filter paper. The filtrate was transferred into a 25mL volumetric flask and diluted with deionized water to calibration mark. For digestion of soil and compost samples, digestion was conducted using procedure similar with that for oven-dried spinach samples using 9 mL of 37% hydrochloric acid and 3 mL of 65% nitric acid (Erdemir and Gucer, 2013).

### 2.6 Elemental analysis

The diluted filtrate was analyzed with inductively coupled plasma optical emission spectrometry (ICP-OES) (PerkinElmer OptimaTM 5300 DV) for the determination of metals and total phosphorous (Yong et al., 2019). The elemental contents in compost, soil, and spinach were calculated based on Eq. (4) (Yong et al., 2017). The total organic carbon (TOC) (% w/w dry basis) was estimated from the LOI based the van Bemmelen factor (1.724) (Eq. (5)) (Yong et al., 2016). Total nitrogen was determined using an elemental analyzer (Thermo).

(4) $$Elemental\;content\;\left(mg/kg\right)\;=\;\frac{\left[Element\right]\times V}{m}$$

(5) $$Total\;organic\;carbon\;=\;\frac{LOI}{1.724}$$

where [Element] is the concentration of element in the diluted filtrate (mg/L); V is the volume of diluted filtrate (L); m is the mass of sample used in the digestion (g). All measurements were conducted in triplicates and the average values were presented. The mean values for soil parameters (soil pH, soil EC, elemental contents) and contents of heavy metals in plant's shoot from SCG-CM and CMC were compared with t-test as available Microsoft EXCEL 2010.

### 2.7 Statistical analysis

All measurements were conducted in triplicates and the average values were presented. The mean values for plant growth (number of leaves, height of stem, width and length of leaves) from SCG-CM and CMC were compared with t-test (paired, two-tail; n = 3) as available in Microsoft EXCEL 2010. The ability for heavy metal in soil and composts-amended soil to migrate to the short of spinach was determined using translocation factor (TF) (Eq. (6)) and accumulation factor (AF) (Eq. (7)) (Usman et al., 2019).

(6) $$TF\;=\;\frac{Concentration\;of\;metal\;in\;shoot}{Concentration\;of\;metal\;in\;root}$$

(7) $$AF\;=\;\frac{Concentration\;of\;metal\;in\;root}{Concentration\;of\;metal\;in\;soil}$$

## 3 Results and discussion

### 3.1 Characterization of SCG-CM, CMC, and soil

Table 1 shows the physico-chemical charateristics for SGC-CM, CMC, and soil. The moisture content for SGC-CM (2.59 ± 0.12% w/w dry basis) is slightly higher than that of CMC (1.22 ± 0.08% w/w dry basis) and soil (1.33 ± 0.02% w/w dry basis). The OM in SGC-CM is higher than those of a finished compost (i.e., 30–70% w/w dry basis). High degree of OM shows a high degree of compost maturity (Moral et al., 2009). The pH of SGC-CM is 8.20 which indicates that the composting process has been completed (Zmora-Nahum et al., 2005).

**Table 1:** Physico-chemical characteristics for SGC-CM, CMC, and soil (% w/w dry Basis).

	SGC-CM	CMC	Soil
Moisture Content (% w/w dry basis)	2.59 ± 0.12	1.22 ± 0.08	1.33 ± 0.02
Organic Matter (% w/w dry basis)	81.42 ± 0.78	72.87 ± 1.06	14.37 ± 0.74
Ash Content (% w/w dry basis)	13.32 ± 0.50	23.38 ± 0.91	72.79 ± 0.82
pH	8.20 ± 0.18	8.16 ± 0.29	7.62 ± 0.004
EC (dS/m)	3.98 ± 0.001	2.35 ± 0.001	0.000428 ± 0.001

**Figure 1:**
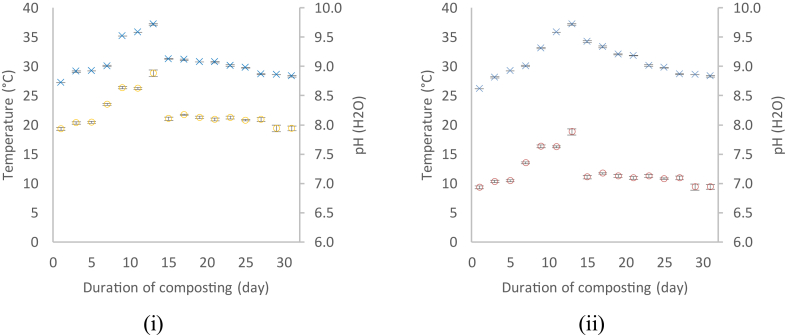
Temperatures (°C) and pH of (i) CMC, and (ii) SGC-CM during composting process (31 days).

Figure 1 shows temperature of SGC and cat manure compost in the process of composting where the temperature was constantly elevating from Day 1 to Day 13. The temperature of the compost started to decline at Day 15 where it gradually on decreasing and became constant around Day 27 where it reached room temperature, 28.7 °C. This shows that the compost has matured completely. The sequence of temperatures show the microbial activity and the occurrence of the composting process (Bernal et al., 2009). The temperature of the compost in this study did not reach the actual temperature range for composting (40–65 °C) (de Bertoldi et al., 1983). This is due to the size of compost which was not that large in quantity.

The concentration of heavy metals in soil, CMC, and SGC-CM are shown in Table 2. The safe amount of heavy metal concentrations were compared with maximum permissible limit in soil by European Union Standard (2002) (Hogg et al., 2002), and also Compost Quality Standard and Guidelines (2000) (Brinton, 2000). The concentration of Cu, 5.34 mg/kg in soil is above the maximum permissible limit in soil by European Union Standard (2002), 2.1 mg/kg. As in CMC, it is shown that the concentration of Cd, 339.6 mg/kg exceeds the maximum permissible limit in compost by Compost Quality Standard and Guidelines (2000), 70 mg/kg which is similar to the concentration of Zn, 1261 mg/kg in SGC-CM which also exceeds the guideline. The amount of Zn in SGC-CM is expected to a higher uptake towards the spinach which is not wanted in the food industry because the high uptake of heavy metals would cause a serious toxicity towards consumers. Although it has an impact upon the growth rate of the spinach.

**Table 2:** Elemental contents (mg/kg dry basis) of SGC-CM, CMC and soil.

Element	Total elemental content (dry weight basis)			EU Standard (2002)	Compost Quality Standard and Guidelines (2000)
	SGC-CM	CMC	Soil		
Zn (mg/kg)	1261.8 ± 0.19∗	2.266 ± 1.23	11.42 ± 2.35	210	210
Cu (mg/kg)	4.66 ± 0.0001	4.44 ± 0.001	5.34 ± 0.001	2.1	5.5
Cd (mg/kg)	50.62 ± 0.0190	339.6 ± 0.07∗	3.44 ± 0.06	98	70
Pb (mg/kg)	3.56 ± 0.0015	13.42 ± 0.01	11.98 ± 0.004	210	70
N (%)	7.0	4.2	1.02		
P (mg/kg)	1209 ± 0.32	1894 ± 0.42	216 ± 0.04		
K (mg/kg)	5438 ± 1.27	2450 ± 0.14	5048 ± 1.05		
TOC (%)	47.23 ± 0.45	42.27 ± 0.61	8.34 ± 0.43		

∗ Exceeds the maximum permissible limit suggested by European Union Standard (2002) and Compost Quality Standard and Guidelines (2000).

### 3.2 The effect of SGC-CM on the growth rate of spinach (*Spinacia oleracea*)

**Figure 2:**
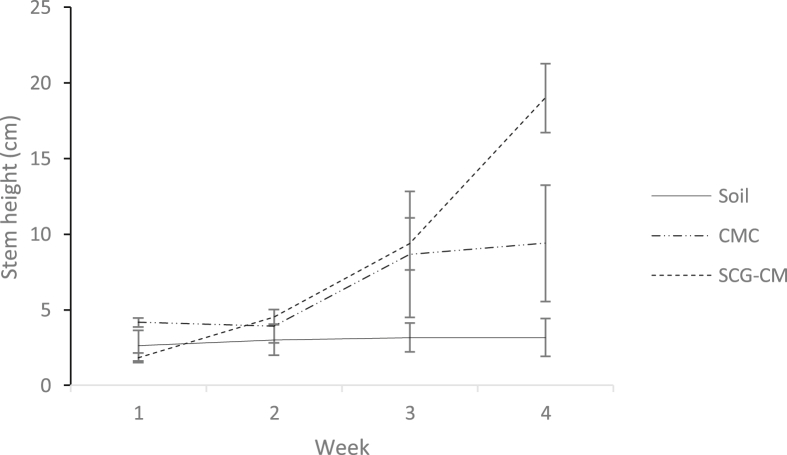
Spinach grown in (i) soil, (ii) CMC, and (iii) SCG-CM on week 4.

**Figure 3:**
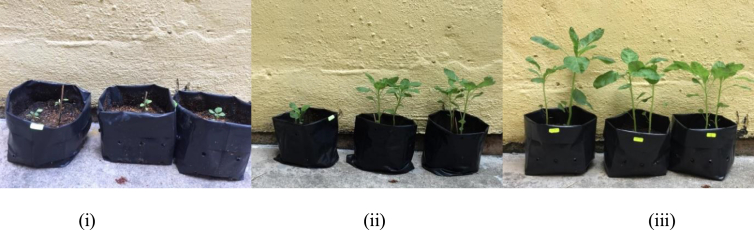
Spinach grown in (i) soil, (ii) CMC, and (iii) SCG-CM on week 4.

**Figure 4:**
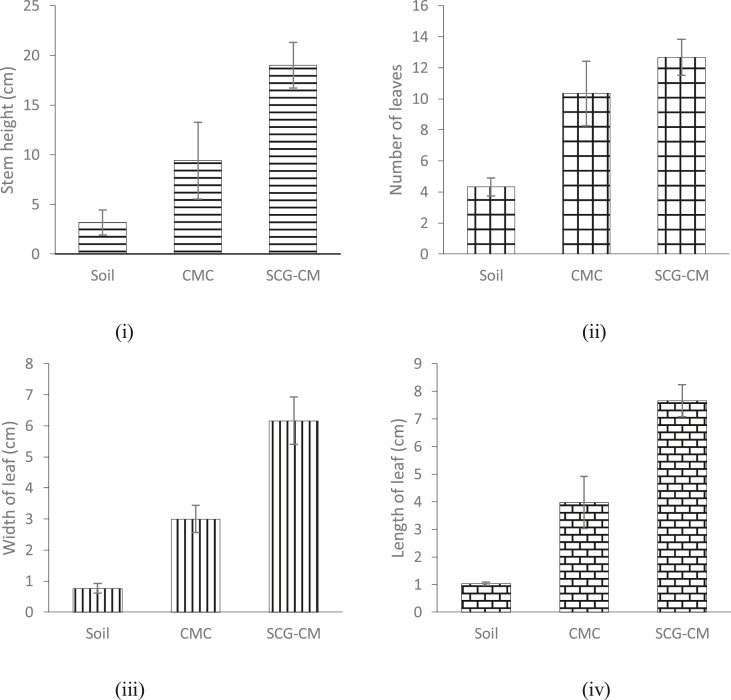
(i) Height (cm), (ii) number of leaves, (iii) width of leaf (cm), and (iv) length of leaf (cm) of spinach grown in soil, CMC-amended soil and SCG-CM-amended soil.

The growth rate of spinach stem height (cm) over four weeks and images of spinach grown in soil, CMC-amended soil, and SCG-CM-amended soil on week four are shown in Figures 2 and 3, respectively. Several growth parameters such as the height, width of leaf, length of leaf and the number of leaves of spinach in different application of composts are shown in Figure 4. The application of SGC-CM produced higher yield compared to CMC and soil. Apart from the number of leaves, all other growth parameters for spinach grown in the SCG-CM-amended soil (i.e., height of stem, width and length of leaf) were significantly greater than those grown in the CMC-amended soil (p < 0.05, n = 3). It is believed that CMC alone will dissolve slowly and does not meet up the yield of vegetables (Oyedeji et al., 2014). One of the factors affecting the growth rate of spinach is the nitrogen uptake. The presence of nitrogen in compost or soil gives an efficient growth rate for the crop. Application of compost with high nitrogen content would give a better yield of plants. In this study, most of the nitrogen comes from CM and CMC. Animal manure does have high amount of nitrogen but the nitrogen forms vary due to the differences in feed, age of animal, bedding material and water intake (Chadwick et al., 2000). Application of CMC is an excellent soil amendment that provides nutrient to grow crops and also improves soil quality (Oyedeji et al., 2014). Moreover, SCG has higher nitrogen content compared to hardwood sawdust. The extra source of nitrogen gives SCG-CM greater nitrogen content, and possibly contributed to the growth of spinach.

### 3.3 The uptake of Zn, Cd, Pb and Cu in the shoot and root of spinach (*Spinacia oleracea*)

Table 3 shows the concentration of Zn, Cd, Pb and Cu in shoot and root of spinach (mg/kg dry weight basis) grown in SCG-CM, CMC, and soil. All heavy metals accumulated in the shoot of CMC were significantly higher than those in the shoot of spinach grown in SCG-CM (p < 0.05). The content of Cu and Cd in the shoot of spinach grown in the CMC-amended soil is more than the permissible limit as compared to maximum permissible limit set by Malaysian Food Act (1983) and Food Regulations 1985 (2006). The source of Cd is probably from the Cd-contaminated CMC (339.6 ± 0.07 mg/kg). The high Cd content in the shoot and root of spinach grown in CMC is expected due to the fact that spinach is a hyperaccumulator of Cd (Rizwan et al., 2017). Copper and zinc were also accumulated in the shoot as it is required by plants as micronutrients (Lu et al., 2015). The higher content of Cu and Zn in the shoot of spinach did not caused growth retardation and may indicate absence of phytotoxicity on spinach. Even though SCG-CM contains higher Zn and Cd than soil, the metal contents in the shoot of spinach grown in SCG-CM and soil were similar and is not statistically significant. This indicates that heavy metals were possibly reacted with compounds in the SCG-CM, causing an immobilization effect and reduced uptake of spinach (Gray and Wise, 2020). Contrary to SCG-CM, CMC.

**Table 3:** Contents of Zn, Cd, Pb, and Cu in shoot and root of spinach (mg/kg) grown in SGC-CM, CMC, and soil (Dry Weight Basis).

Element	Total content (mg/kg, dry weight basis)						Malaysian Food Act 1983 (The Attorney Generals's Chamber of Malaysia, 2006)	FAO/WHO (FAO/WHO, 2011)
	SGC-CM		CMC		Soil			
	Shoot	Root	Shoot	Root	Shoot	Root		
Zn	0.83 ± 0.05	1.39 ± 1.04	22.53 ± 2.57	23.53 ± 2.50	2.30 ± 0.14	2.00 ± 0.32	40	20
Cu	1.11 ± 0.24	0.90 ± 0.12	9.60 ± 1.06	8.73 ± 0.23	0.87 ± 0.04	0.85 ± 0.02	30	30
Cd	0.25 ± 0.06	0.20 ± 0.01	2.80 ± 0.87∗	2.20 ± 0.00	0.23 ± 0.02	0.23 ± 0.01	2	0.2
Pb	0.67 ± 0.10	0.55 ± 0.01	6.40 ± 1.25	5.60 ± 0.20	0.63 ± 0.09	0.54 ± 0.00	2	0.3

∗ Exceeded permissible limit by Malaysian Food Act (1983) and Food Regulations 1985 (2006).

### 3.4 Translocation factor

Table 4 shows the TF and AF values for Zn, Cu, Cd and Pb for spinach grown in soils amended with SGC-CM and CMC. Spinach grown on pristine soil has TF value greater than 1 for Cd. This finding is consistent with those from literature, whereby, spinach is known to as hyperaccumulator for Cd and does not show growth retardation at low soil Cd content (<20 mg/kg) (Salaskar et al., 2011). However, a decrease of TF values for Cd was observed for both SGC-CM and CMC-amended soils. This result shows that soil amendment with SCG-CM and CMC decreased the tendency of spinach to translocate Zn to shoot tissue, while translocation of Cu was slightly increased. The slightly alkaline nature for both SCG-CM (8.20 ± 0.18) and CMC (8.16 ± 0.29) may have increased pH of pristine soil (7.62 ± 0.004), causing precipitation of available species of heavy metals and decreased Cd uptake by spinach. All AF values except for Zn (2.15) and Cu (1.65) for CMC were lower than 1. This indicates that application of SCG-CM did not enhance accumulation of heavy metals into the root section of spinach. Furthermore, the large polyphenol compounds are relatively non-polar and may not be extracted by water during the coffee brewing process. During composting process with CM and chicken manure, the increasing pH values may deprotonate the residual polyphenols to form phenolate group and facilitates the chelation of water-soluble heavy metals in SCG. Immobilization of soluble heavy metals in SCG reduces the availability and contributed to a low uptake by spinach. The phytotoxic effect of polyphenols in SCG-CM is not evident as shown in Figure 2 and Figure 3. Co-composting of SCG with CM may have alleviate the phytotoxicity of polyphenols and improved plant growths (Yamane et al., 2014). Besides that, the negatively-charged surface of SCG may fix N compounds and help reduce the loss of N from CM.

**Table 4:** Translocation factor (TF) and accumulation factor (AF) values for Zn, Cd, Pb, and Cu for spinach grown in SGC-CM, CMC, soil.

Element	TF			AF		
	SCG-CM	CMC	Soil	SCG-CM	CMC	Soil
Zn	0.60	0.96	1.15	0.02	2.15	0.18
Cu	1.23	1.10	1.02	0.17	1.65	0.16
Cd	1.25	1.27	1.00	0.03	0.11	0.07
Pb	1.22	1.14	1.17	0.05	0.46	0.05

## 4 Conclusion

This study concludes that the application of the SGC-CM for growing spinach has a better crop yield than that of CMC as indicated by the greater height of stem, width of leaf, length of leaf and number of leaves. However, the uptake of nutrients (K & P) by spinach in the soil amended with CMC is higher than that in the SCG-CM-amended soil. Similarly, the uptake of heavy metals such as Zn, Cu, Cd and Pb in spinach is decreased when soil is amended with SGC-CM and is safe to be consumed as required by the Malaysian Food Act 1983 and Food Regulations 1985 (2006). The immobilization effect of heavy metals was probably due to chelation of polyphenols compounds in the SCG. This study has shown promising result on the use of SCG for stabilizing animal manure for growing food crop. Further study should be carried on the long-term application of SCG-CM on soil properties, plant growth, and potential impact on soil microbial community. Further analysis on the biocidal effect of co-composting with SGC on the pathogens in CM is also crucial for ensuring safe use of cat manure and reducing the health risk associated with the pathogenic microbe in animal manure.

## Declarations

### Author contribution statement

Siti Nurathirah Kamaliah Mohd Noor Keeflee: Conceived and designed the experiments; Performed the experiments; Analyzed and interpreted the data; Wrote the paper.

Wan Nur Azra Wan Mohd Zain: Performed the experiments.

Muhammad Nuruddin Mohd Nor: Analyzed and interpreted the data.

Nurul’ Ain Jamion: Analyzed and interpreted the data; Contributed reagents, materials, analysis tools or data.

Soon Kong Yong: Conceived and designed the experiments; Analyzed and interpreted the data; Contributed reagents, materials, analysis tools or data; Wrote the paper.

### Funding statement

This work was supported by the 10.13039/501100003093Ministry of Higher Education, Malaysia (FRGS/1/2017/WAB05/UITM/03/2) and 10.13039/501100004625Universiti Teknologi MARA for providing the research facility.

### Competing interest statement

The authors declare no conflict of interest.

### Additional information

No additional information is available for this paper.
